# Bipolar and Complementary Resistive Switching Characteristics and Neuromorphic System Simulation in a Pt/ZnO/TiN Synaptic Device

**DOI:** 10.3390/nano11020315

**Published:** 2021-01-27

**Authors:** Sobia Ali Khan, Geun Ho Lee, Chandreswar Mahata, Muhammad Ismail, Hyungjin Kim, Sungjun Kim

**Affiliations:** 1 School of Electronics Engineering, Chungbuk National University, Cheongju 28644, Korea; sobiaali717@ymail.com (S.A.K.); chandreswar@gmail.com (C.M.); 2 Department of Electronic Engineering, Inha University, Incheon 22212, Korea; hhhh1594@naver.com; 3 Division of Electronics and Electrical Engineering, Dongguk University, Seoul 04620, Korea; ismailmalikbzu10@gmail.com

**Keywords:** resistive switching, memristor, synaptic device, neural network simulation, X-ray photoelectron spectroscopy, neuromorphic system

## Abstract

In this work, a ZnO-based resistive switching memory device is characterized by using simplified electrical conduction models. The conventional bipolar resistive switching and complementary resistive switching modes are accomplished by tuning the bias voltage condition. The material and chemical information of the device stack including the interfacial layer of TiON is well confirmed by transmission electron microscopy (TEM) and X-ray photoelectron spectroscopy (XPS) analysis. The device exhibits uniform gradual bipolar resistive switching (BRS) with good endurance and self-compliance characteristics. Moreover, complementary resistive switching (CRS) is achieved by applying the compliance current at negative bias and increasing the voltage at positive bias. The synaptic behaviors such as long-term potentiation and long-term depression are emulated by applying consecutive pulse input to the device. The CRS mode has a higher array size in the cross-point array structure than the BRS mode due to more nonlinear I–V characteristics in the CRS mode. However, we reveal that the BRS mode shows a better pattern recognition rate than the CRS mode due to more uniform conductance update.

## 1. Introduction

Resistive random-access memory (RRAM) is one of the strongest candidates for the next-generation nonvolatile memory technology due to low power consumption [1], fast switching speed [2], good complementary metal–oxide–semiconductor (CMOS) compatibility [3,4,5], and high scalability [6]. Until now, RRAM has been developed to focus on its applications such as high-density memory, embedded memory, and neuromorphic system. For storage class memory and embedded memory applications, the fast switching speed of RRAM is attractive. However, large variability and crosstalk issues are big obstacles to commercialization of RRAM [7,8,9]. Among a lot of resistor materials, the metal oxides such as HfO_2_, TaO*_x_*, TiO_2_, ZrO, and ZnO attract huge interest due to good resistive switching performance such as endurance, retention, high switching speed, and relatively good variability [10]. ZnO especially is well-known as an n-type semiconductor material, and oxygen vacancies can be controlled by the applied bias [11]. The various resistive switching behaviors have been reported depending on the deposition stack and switching operation conditions. For example, the unipolar resistive switching (URS) and homogeneous BRS were controllable by the compliance current in a Pt/ZnO/Pt device [12]. Moreover, the CRS behavior was observed in a bilayered TiN/MgZnO/ZnO/Pt stack with asymmetric electrodes [13]. The endurance of Pt/ZnO/Pt is up to the 10^6^ cycle and good retention is reported for several ZnO-based RRAM devices [14]. The on/off ratio can be maximized when using Ag and Cu top electrodes with a sharp I–V slope in a ZnO-based RRAM device. The on/off ratio of 10^7^ was reported for an Ag/ZnO:Mn/Pt device [15].

Recently, there have been many efforts to use RRAM as a synapse device for hardware neuromorphic systems [16,17,18,19,20,21,22,23,24,25,26,27,28,29,30,31,32]. Multiple conductance can be achieved by using gradual set and reset switching behaviors and low-power operation is one of the strong points of RRAM. Even though a lot of ZnO-based RRAM devices have been reported for high-density memory application, ZnO-based RRAM needs to be improved more using the materials and electrical engineering for neuromorphic systems. Gradual conductance modulation especially is very important to implement a synaptic device.

In this work, we characterize gradual BRS and CRS from a Pt/ZnO/TiN device by controlling operation polarity and compliance current. The gradual set and reset switching in CRS were rarely reported in the past. Here, we demonstrate potentiation and depression in both BRS and CRS for a neuromorphic system. Moreover, we conducted a comparison study on pattern recognition using the Modified National Institute of Standards and Technology (MNIST) data in a single-layer neural network when using gradual BRS and CRS modes. We conducted pattern recognition considering the variation that had rarely been reported in the literature.

## 2. Materials and Methods

The sample preparation process sequence is as follows. Firstly, a 10 nm-thick ZnO film was deposited by radio frequency (RF) magnetron sputtering at room temperature on a TiN/SiO_2_/Si substrate. High-purity argon (Ar: 99.999%) and oxygen (O_2_: 99.999%) with the Ar/O_2_ ratio of 6/14 standard cubic centimeters per minute (SCCM) were flowed into the chamber during the deposition. During ZnO sputtering as a switching layer, RF power and working pressure were fixed at 100 W and 5 mTorr, respectively. Finally, Pt top electrode was deposited on the ZnO film by direct current (DC) magnetron sputtering at room temperature (RT) to form a Pt/ZnO/TiN RRAM device. A shadow mask with a 100 µm diameter was used to pattern the top electrodes. The electrical properties were characterized in the DC mode using a Keithley 4200-SCS semiconductor parameter analyzer and in the pulse mode using a 4225-PMU ultrafast module. For all electrical measurements, external bias was applied to the Pt top electrode (TE), while the TiN bottom electrode (BE) was grounded.

## 3. Results and Discussion

**Figure 1:**
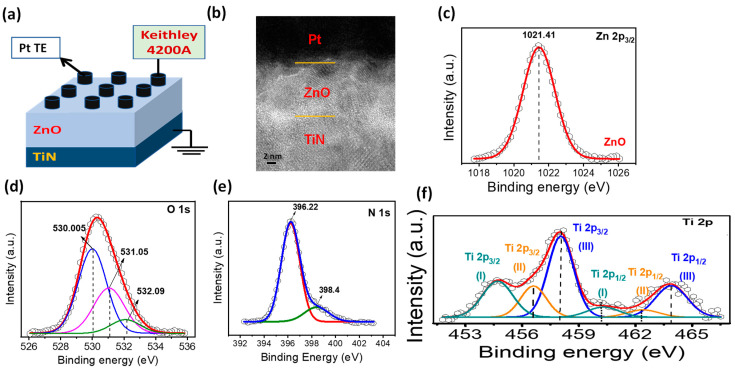
(**a**) Schematic diagram of Pt/ZnO/TiN. (**b**) Cross-sectional TEM image of a single-layer device; XPS spectra of (**c**) Zn 2p, (**d**) O 1s, (**e**) N 1s, (**f**) Ti 2p.

**Figure 2:**
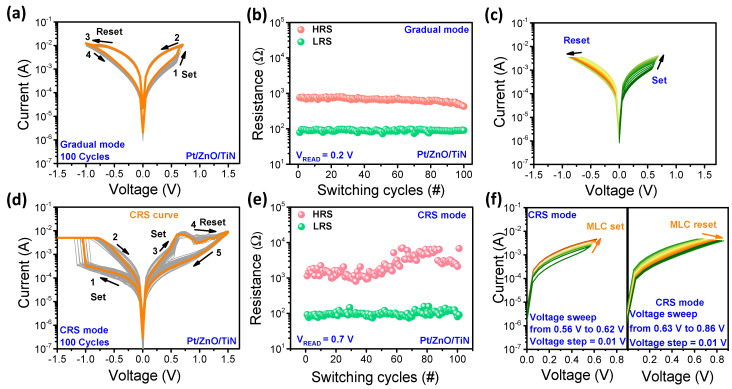
Gradual set and reset bipolar mode of the Pt/ZnO/TiN device. (**a**) Typical CC-free I–V curve (100 consecutive cycles). (**b**) Endurance according to the I–V data. (**c**) Multilevel switching of set and reset with gradually increasing and decreasing behavior. (**d**) I–V curves for 100 consecutive cycles with CC of 5 mA. (**e**) Endurance of the CRS mode. (**f**) Multilevel switching by increasing and decreasing behavior of CRS in the positive bias region.

Figure 1a demonstrates the schematic structure of a Pt/ZnO/TiN device. The ZnO film sandwiched between the Pt and TiN electrode layers is observed in the cross-sectional TEM image of the Pt/ZnO/TiN device. The chemical nature of different elements present, the ZnO/TiN interfacial layer, as well as the ZnO layer are characterized by XPS. The Zn 2p, O 1s, and Ti 2p XPS peaks were analyzed as shown in Figure 1c–e. Figure 1c presents the fitted Zn 2p spectra for the top ZnO layer taken. The peak binding energy of Zn 2p in ZnO was located at 1021.41 eV, which is in good agreement with the reported value for bulk ZnO [29]. The XPS peak for metallic Zn at low binding energy was not found, which further confirms that Zn exists only in the oxidized state [33]. Figure 1d displays the XPS peak fitting of O 1s in bulk ZnO. The O 1s spectra were found composed of three peaks centered at 530.005, 531.05, and 532.09 eV, respectively, with three best-fitted curves. The lowest binding energy peak at 530.005 eV was related to ZnO in the bulk [34]. Other peaks at a binding energy of 531.05 eV and 532.09 eV were related to O^2−^ ions in the oxygen-deficient regions within the matrix of ZnO [35]. The binding energy at 531.05 eV attributes the chemical bonding of oxygen with Ti and causes ZnOTi due to the interface layer at the TiN bottom electrode [36]. The highest peak of O 1s is at-tributed to TiO [37]. Figure 1e shows XPS spectra of N 1s from the ZnO/TiN stack. The Main peak was centered at 396.22 eV for TiN and another peak was also observed at 398.4 eV for the TiON layer that is the surface oxidation of the TiN bottom electrode [38]. To analyze the interface’s chemical composition between ZnO/TiN, Ti 2p doublet was fitted with Gaussian–Lorentzian function and the Shirley background correction in Figure 2f. Ti 2p spectra were deconvoluted, as it exposed the broad shoulder peaks at both the Ti 2p_3/2_ and Ti 2p_1/2_ doublets as shown in Figure 1e. For TiO bonding, two peaks for Ti 2p_3/2_ and Ti 2p_1/2_ were located at 458.1 eV and 463.9 eV with the energy split-up of 5.8 eV, which is similar to the reported result [39]. All Ti 2p spectra were fitted with three different doublets with peak positions of 458.1 eV and 463.9 eV for TiO, 456.6 eV and 462.4 eV for TiON, and the lowest binding energies were found for TiN at 454.6 eV and 460.4 eV [40].

Figure 2a shows I–V curves of the Pt/ZnO/TiN device in the BRS mode. Firstly, we applied voltage without current compliance (CC) by using its self-compliance characteristics. The device showed a gradual set and reset behavior with a stable on/off ratio up to 100 consecutive cycles. Initially, the device was in the high-resistance state (HRS). After applying the positive voltage of 0.7 V, the state of the device turned to low-resistance state (LRS) by the formation of a conducting filament (CF). Afterwards, LRS returned to HRS when the negative bias was applied. Each cycle underwent the sequence of sweep from 0 V to 0.7 V for the set process and then from 0 V to −1 V for the reset process by applying different polarities to the Pt top electrode. This test was carried out with different cells of the device and the on/off ratio on each cell was almost similar showing bipolar-type analog resistive switching. The endurance test was conducted with 100 consecutive DC sweeps (Figure 2a). Figure 2b shows endurance with the variation of current at a read voltage of 0.2 V with a decreasing trend of LRS after about 85 cycles. To demonstrate the multilevel characteristics, DC sweep was operated with a different voltage range. The set stop voltage was from 0.57 V to 0.69 V with the step of 0.01 V, resulting in the different conductance states during the set process. The device finally reached LRS after each sweep was added. The reset process occured by using the voltage sweep from −0.68 V to −0.87 V with the step of −0.01 V, which resulted in a decreasing trend with different conductance levels.

Next, we focused on the CRS in the positive region when applying different bias conditions (negative set and positive reset). Figure 2d shows the typical I–V curves observed by DC sweep in the Pt/ZnO/TiN device. The set and reset processes occurred at negative bias and positive bias, respectively. The set process at negative bias needs CC to protect the device from strong breakdown. It is noted that the reset is completed after an additional set process at positive bias, which can be classified as CRS. The low current at negative bias after the set process requires an additional set process at positive bias. Nonlinear I–V characteristics are achieved at positive bias due to unique CRS. To test reliability and reproducibility of the CRS mode of the Pt/ZnO/TiN device, the endurance and retention were checked (Figure 2e). One hundred consecutive cycles were observed with quite stable LRS and slight fluctuations in HRS were read at 0.7 V. LRS was almost stable at about 10^2^ Ω, but HRS was initially about 2 × 10^3^ Ω and after 50 cycles it fluctuated and tended to slightly increase up to 7 × 10^3^ Ω. Moreover, the CRS behavior with multilevel cells (MLC) was demonstrated in the positive region as shown in Figure 2f. The device showed the conversion of HRS to LRS for repetitive sweep by controlling stop voltages from 0.56 V to 0.62 V with the step of 0.01 V. As we increased the stop voltage to 0.63 V, the device gradually started to change its state to HRS with increasing voltage. The current was gradually decreased by incrementally applying stop voltage from 0.63 V to 0.86 V. The repetitive set process gradually increased the current level, causing gradual growth of the filament. On the other hand, in the reset process, resistance gradually increased with each sweep due to the recombination of oxygen ions with vacancies for the gradual reset process.

**Figure 3:**
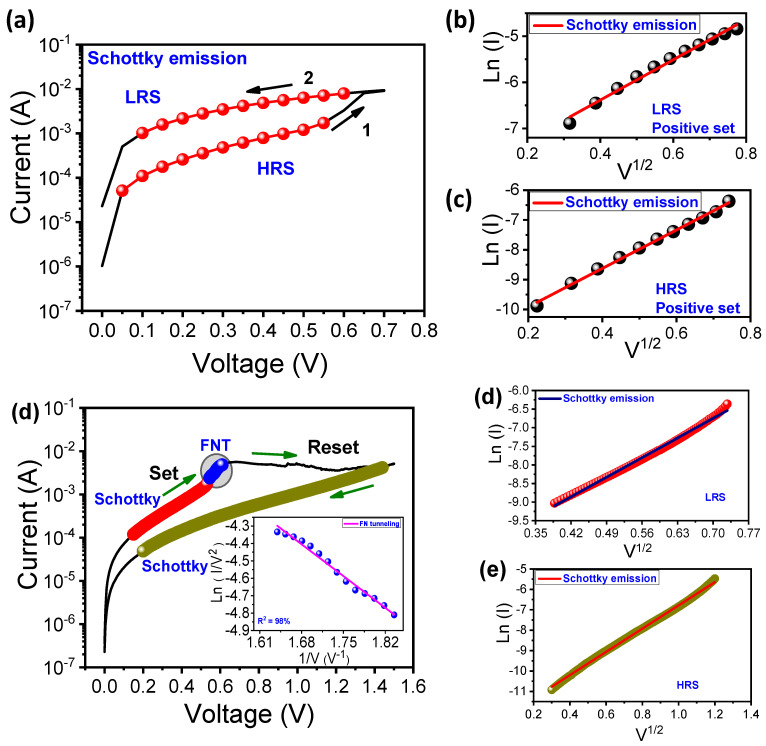
I–V fitting of the Pt/ZnO/TiN device for conduction mechanisms: (**a**) I–V curves for the Schottky emission in LRS and HRS for BRS; (**b**,**c**) Ln(I) versus V^1/2^ fitting for LRS and HRS; (**d**) I–V curves in LRS and HRS for CRS; (**d**,**e**) Ln(I) versus V^1/2^ fitting for LRS and HRS.

Next, we studied the conduction mechanism of the Pt/ZnO/TiN device in the BRS and CRS modes. Figure 3a shows the I–V curves in LRS and HRS for the Schottky emission. The curves of Ln(I) versus V^1/2^ were well-matched with both LRS and HRS for Schottky emission in Figure 3b and Figure 3c, respectively. This indicates that electron conductions in HRS and LRS are limited by the barriers between Zn and the electrodes for BRS [41]. Similarly, LRS in the low-voltage region (~0.545 V) and HRS are well-matched with Ln(I) versus V^1/2^ fitting (Figure 3c–e). However, Fowler–Nordheim tunneling (FNT) can occur in the high-voltage region (from 0.545 V to 0.61 V), which is supported by ln(I/V^2^) versus 1/V fitting (inset of Figure 3c). The current density of FNT is dependent on the electric field. Electrons easily tunnel through a triangular barrier in the presence of a high electric field [41].

**Figure 4:**
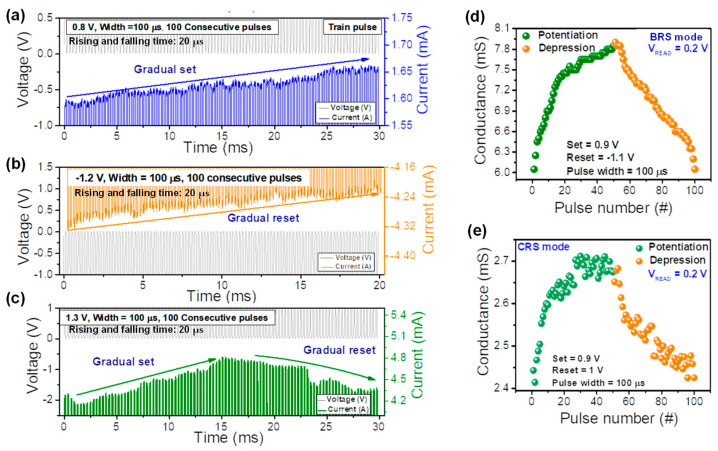
Synaptic plasticity of the Pt/ZnO/TiN device. Pulse train of (**a**) set and (**b**) reset for the BRS mode and (**c**) set and reset for the CRS mode; potentiation and depression of (**d**) the BRS mode and (**e**) the CRS mode.

Next, we study synaptic characteristics of the Pt/ZnO/TiN device for the hardware-based neuromorphic system. As the first step, the repetitive electrical pulses were applied to scan the current and conductance levels of the Pt/ZnO/TiN device. The response highly depends upon the number of pulses when the training pulse is applied to the device. For low-amplitude pulses, many pulses are applied to the device and small pulses are needed to reach target conductance. The characteristics of bipolar analog resistive switching in the pulse mode were investigated via pulse trains. Figure 4a,b presents the gradual set and reset process by identical pulse responses. Figure 4a shows the gradual set process at the train pulse voltage of 100 consecutive cycles (pulse amplitude: 0.8 V; pulse width: 100 µs), leading to correspondence of the output current to the gradually increasing current. Figure 4b shows the gradual reset characteristics by 100 consecutive pulse inputs (pulse amplitude: −1.2 V; pulse width: 100 µs). As a pulse is applied to the Pt/ZnO/TiN device, the absolute value of the current generally tends to decrease. These characteristics are similar to multilevel BRS as confirmed by the DC sweep mode in Figure 2c. Figure 4c presents the training pulse of 100 consecutive pulses in the CRS mode. To control and clarify the CRS mode (current increase and decrease) by multiple pulses, we applied the pulse amplitude of 1.3 V with the pulse width of 100 µs. We found both gradual set and reset during 100 cycles. Initially, the current started to increase gradually, but after about half the time (0.014 s), the current decreasing behavior was observed. It was noted that the controllable gradual set and reset were achieved at identical pulse inputs.

Next, the potentiation and depression in biological synapses were mimicked for the Pt/ZnO/TiN device in both the BRS and CRS modes. The conductance values were extracted from the read voltage of 0.2 V to use vector multiplication in an artificial memristor array and neurons. Figure 4d shows the potentiation and depression characteristics for the BRS mode. The conductance was gradually modulated by the series of voltage pulses (pulse amplitude: 0.9 V, pulse width: 100 µs, raise and fall time of the pulses: 20 µs). Furthermore, the conductance was gradually decreased by the pulses (pulse amplitude: −1.1 V, pulse width: 100 µs, raise and fall time of the pulses: 20 µs). Moreover, we obtained the potentiation and depression characteristics in the CRS mode by consecutively applying 100 pulses. The voltage amplitudes were 0.9 V and 1 V for the potentiation. From approximately the 40th pulse, the variation in conductance became severe and saturated. Note that the potentiation and depression characteristics in the CRS mode were determined using an updated voltage amplitude-controlled conductance method.

**Figure 5:**
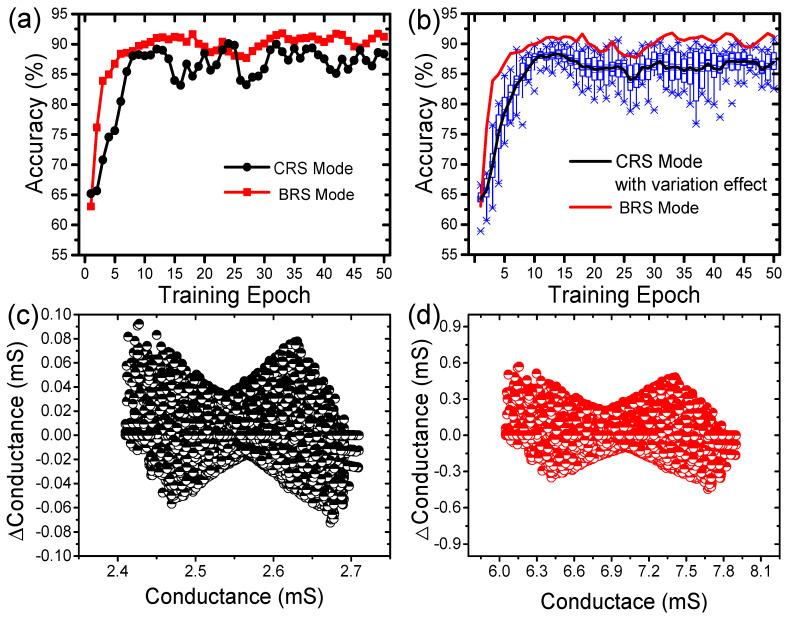
Neuromorphic system depending on the switching mode: recognition accuracy (**a**) without and (**b**) with considering the variation of the learning property; distribution of weight for (**c**) the CRS mode and (**d**) the BRS mode.

In order to verify the effect of switching mode on the performance of a neuromorphic system composed of Pt/ZnO/TiN devices, pattern recognition simulation with a single-layer (784 × 10) neural network was conducted using the Modified National Institute of Standards and Technology (MNIST) data with 60,000 training and 10,000 test images. Here, we developed a device model for both switching modes with learning characteristics and assumed that every learning event occurred after each batch run. Figure 5a shows the accuracy depending on the switching mode without variation effects. Even without considering the variation characteristics, the pattern recognition accuracy with the CRS mode was unstable and low due to nonlinear weight update behaviors. This is because nonlinearity induces abrupt weight updates, resulting in fluctuating training characteristics according to the number of training epochs. In addition, when the variation effects are considered, the performance of pattern recognition with the CRS mode has a large fluctuation with maximum 20% of accuracy difference at the same epoch as shown in Figure 5b because the learning property of the CRS mode is more unpredictable than that of the CRS mode in terms of cycle-to-cycle variation. This implies that the neuromorphic system run with the BRS mode is more promising than the CRS mode with regard to the average and uniform performance. Figure 5c,d shows the distribution of weight changes according to the current conductance value of all epochs for the CRS mode and the BRS mode, respectively. It confirms that the characteristics of weight update with the CRS mode are quite abrupt, leading to large and fluctuating degradation of recognition accuracy even after dozens of epochs.

## 4. Conclusions

In summary, the chemical and material properties of the interfacial layer of TiON as well as each layer in the Pt/ZnO/TiN device were analyzed by XPS. BRS and CRS in the DC sweep mode were characterized by voltage polarity. Moreover, multilevel switching was demonstrated by controlling the set and reset stop voltages. Next, the transient characteristics and conductance update were investigated using repetitive pulses. The potentiation and depression were well-emulated for BRS and CRS. The BRS mode showed a better conductance update with lower variation than the CRS mode. Finally, the artificial potentiation and depression characteristics of both the BRS and CRS modes in the Pt/ZnO/TiN device were evaluated through neuromorphic simulation.

## References

[1] Ryu H., Kim S. (2020). Self-Rectifying Resistive Switching and Short-Term Memory Characteristics in Pt/HfO_2_/TaO*_x_*/TiN Artificial Synaptic Device. Nanomaterials.

[2] Kim H.D., An H.M., Lee E.B., Kim T.G. (2011). Stable Bipolar Resistive Switching Characteristics and Resistive Switching Mechanisms Observed in Aluminum Nitride-based ReRAM Devices. IEEE Trans. Electron. Dev..

[3] Choi J., Kim S., Zeng F. (2020). Coexistence of Long-Term Memory and Short-Term Memory in an SiN*_x_*-Based Memristor. Phys. Status. Solid RRL.

[4] Choi J., Kim S. (2020). Improved Stability and Controllability in ZrN-Based Resistive Memory Device by Inserting TiO_2_ Layer. Micromachines.

[5] Tikhov S.V., Gorshkov O.N., Antonov I.N., Tetelbaum D.I., Mikhaylov A.N., Belov A.I., Morozov A.I., Karakolis P., Dimitrakis P. (2018). Behavioral Features of MIS Memristors with a Si_3_N_4_ Nanolayer Fabricated on a Conductive Si Substrate. Semiconductors.

[6] Maikap S., Banergee W. (2020). In Quest of Nonfilamentary Switching: A Synergistic Approach of Dual Nanostructure Engineering to Improve the Variability and Reliability of Resistive Random-Access-Memory Devices. Adv. Electron. Mater..

[7] Choi J., Kim S. (2020). Nonlinear Characteristics of Complementary Resistive Switching in HfAlO*_x_*-Based Memristor for High-Density Cross-Point Array Structure. Coatings.

[8] Wu L., Liu H., Lin J., Wang S. (2020). Self-Compliance and High Performance Pt/HfO*_x_*/Ti RRAM through annealing. Nanomaterials.

[9] Linn E., Rosezin R., Kügeler C., Waser R. (2010). Complementary resistive switches for passive nanocrossbar memories. Nat. Mater..

[10] Lee M.-J., Lee C.B., Lee D., Lee S.R., Channg M., Hur J.H., Kim C.-J., Seo D.H., Kim Y.B., Kim C.J. (2011). A fast, high-endurance and scalable non-volatile memory device made from asymmetric Ta_2_O_5−*x*_/TaO_2−*x*_ bilayer structures. Nat. Mater..

[11] Simanjuntak F.M., Panda D., Wei K.H., Tseng T.Y. (2016). Status and Prospects of ZnO-Based Resistive Switching Memory Devices. Nanoscale Res. Lett..

[12] Huang C.H., Huang J.S., Lai C.C., Huang H.W., Lin S.J., Chueh Y.L. (2013). Manipulated transformation of filamentary and homogeneous resistive switching on ZnO thin film memristor with controllable multistate. ACS Appl. Mater. Interfaces.

[13] Chen X., Hu W., Wu S., Bao D. (2014). Complementary switching on TiN/MgZnO/ZnO/Pt bipolar memory devices for nanocrossbar arrays. J. Alloys Compd..

[14] Chiu F.C., Li P.W., Chang W.Y. (2012). Reliability characteristics and conduction mechanisms in resistive switching memory devices using ZnO thin films. Nanoscale Res. Lett..

[15] Yang Y.C., Pan F., Liu Q., Liu M., Zeng F. (2019). Fully room-temperaturefabricated nonvolatile resistive memory for ultrafast and high-density memory application. Nano Lett..

[16] Emelyanov A.V., Nikiruy E.K., Serenko A.V., Sitnikov A.V., Presnyakov M.Y., Rybka R.B., Sboev A.G., Rylkov V.V., Kashkarov P.K., Kovalchuk M.V. (2019). Self-adaptive STDP-based learning of a spiking neuron with nanocomposite memristive weights. Nanotechnology.

[17] Mikhaylov A., Pimashkin A., Pigareva Y., Gerasimova S., Gryaznov E., Shchanikov S., Zuev A., Talanov M., Lavrov I., Demin V. (2020). Neurohybrid Memristive CMOS-Integrated Systems for Biosensors and Neuroprosthetics. Front. Mol. Neurosci..

[18] Kuzum D., Yu S., Wong H.-S.P. (2013). Synaptic electronics: Materials, devices and applications. Nanotechnology.

[19] Ielmini D., Wong H.-S.P. (2018). In-memory computing with resistive switching devices. Nat. Electron..

[20] Lin P., Li C., Wang Z., Li Y., Jiang H., Song W., Rao M., Barnell M., Zhuo Y., Upadhyay N.K. (2020). Three-dimensional memristor circuits as comple neural networks. Nat. Electron..

[21] Kim C.-H., Lim S., Woo S.Y., Kang W.M., Seo Y.-T., Lee S.T., Lee S., Kwon D., Oh S., Noh Y. (2018). Emerging memory technologies for neuromorphic computing. Nanotechnology.

[22] Ryu H., Choi J., Kim S. (2020). Voltage Amplitude-Controlled Synaptic Plasticity from Complementary Resistive Switching in Alloying HfO*_x_* with AlO*_x_*-Based RRAM. Metals.

[23] Ryu H., Kim S. (2020). Synaptic Characteristics from Homogeneous Resistive Switching in Pt/Al_2_O_3_/TiN Stack. Nanomaterials.

[24] Wang R., Shi T., Zhang X., Wang W., Wei J., Lu J., Zhao X., Wu Z., Cao R., Long S. (2018). Bipolar Analog Memristors as Artificial Synapses for Neuromorphic Computing. Materials.

[25] Jang J.T., Min J., Hwang Y., Choi S.-J., Kim D.M., Kim H., Kim D.H. (2020). Digital and analog switching characteristics of InGaZnO memristor depending on top electrode material for neuromorphic system. IEEE Access.

[26] Kim T.-H., Nili H., Kim M.-H., Min K.K., Park B.-G., Kim H. (2020). Reset-voltage-dependent precise tuning operation of TiO*_x_*/Al_2_O_3_ memristive crossbar array. Appl. Phys. Lett..

[27] Prezioso M., Mahmoodi M., Merrikh-Bayat F., Nili H., Kim H., Vincent A., Strukov D. (2018). Spike-timing-dependent plasticity learning of coincidence detection with passively integrated memristive circuits. Nat. Commun..

[28] Kim S., Kim T.-H., Kim H., Park B.-G. (2020). Current suppressed self-compliance characteristics of oxygen rich TiO*_y_* inserted Al_2_O_3_/TiO*_x_* based RRAM. Appl. Phys. Lett..

[29] Xu H.Y., Liu Y.C., Ma J.G., Luo Y.M., Lu Y.M., Shen D.Z., Zhang J.Y., Fan X.W., Mu R. (2004). Photoluminescence of F-Passivated ZnO Nanocrystalline Films Made from Thermally Oxidized ZnF^2^ Films. J. Phys. Condens. Matter.

[30] Shi Z., Ha S.D., Zhou Y., Schoofs F., Ramanathan S. (2013). A correlated nickelate synaptic transistor. Nat. Commun..

[31] Ryu J.H., Kim S. (2020). Artificial synaptic characteristics of TiO_2_/HfO_2_ memristor with self-rectifying switching for brain-inspired computing. Chaos Solitons Fractals.

[32] Ryu J.H., Mahata C., Kim S. (2021). Long-term and short-term plasticity of Ta_2_O_5_/HfO_2_ memristor for hardware neuromorphic application. J. Alloy. Compd..

[33] Chen M., Wang X., Yu Y.H., Pei Z.L., Bai X.D., Sun C., Huang R.F., Wen L.S. (2000). X-Ray Photoelectron Spectroscopy and Auger Electron Spectroscopy Studies of Al-Doped ZnO Films. Appl. Surf. Sci..

[34] Zhang X.T., Liu Y.C., Zhang J.Y., Lu Y.M., Shen D.Z., Fan X.W., Kong X.G. (2003). Structure and Photoluminescence of Mn-Passivated Nanocrystalline ZnO Thin Films. J. Cryst. Growth.

[35] Fan J.C., Goodenough J.B. (1977). X-Ray Photoemission Spectroscopy Studies of Sn-Doped Indium-Oxide Films. J. Appl. Phys..

[36] Bertoti I., Mohai M., Sullivan J., Saed S.-A. (1995). Surface Characterisation of Plasma-Nitrided Titanium: An XPS Study. Appl. Surf. Sci..

[37] Zhou X., Jin B., Zhang S., Wang H. (2012). Preparation of Boron and Phosphor Co-Doped TiO_2_ Nanotube Arrays and Their Photoelectrochemical Property. Electrochem. Commun..

[38] Duta L., Stan G.E., Popa A.C., Husanu M.A., Moga S., Socol M., Zgura I., Miculescu F., Urzica I., Popescu A.C. (2016). Thickness Influence on In Vitro Biocompatibility of Titanium Nitride Thin Films Synthesized by Pulsed Laser Deposition. Materials.

[39] Mahata C., Das T., Mallik S., Hota M.K., Maiti C.K. (2011). Chemical Bonding States of Plasma Nitrided High-k/Ge Gate Stack. Electrochem. Solid State Lett..

[40] Hasegawa G., Kitada A., Nakanishi K., Kobayashi Y. (2014). Impact of Electrolyte on Pseudocapacitance and Stability of Porous Titanium Nitride (TiN) Monolithic Electrode Electrodeposition of Metals with Low Negative Potentials View Project Copper Electrorefining View Project. J. Electrochem. Soc..

[41] Chang Y.F., Fowler B., Chen Y.C., Chen Y.T., Wang Y., Xue F., Zhou F., Lee J.C. (2014). Intrinsic SiO*_x_*-based unipolar resistive switching memory. II. Thermal effects on charge transport and characterization of multilevel programing. J. Appl. Phys..

